# Supplementary material for: Proteomic Analysis of Arsenic Resistance during Cyanide Assimilation by Pseudomonas pseudoalcaligenes CECT 5344
Source: Int J Mol Sci. 2023 Apr 13;24(8):7232. doi: 10.3390/ijms24087232 (PMC10138600; doi:10.3390/ijms24087232)
Supplement: Supplementary file 1 [file ijms-24-07232-s001.zip › ijms-2303921-supplementary figures.pdf]

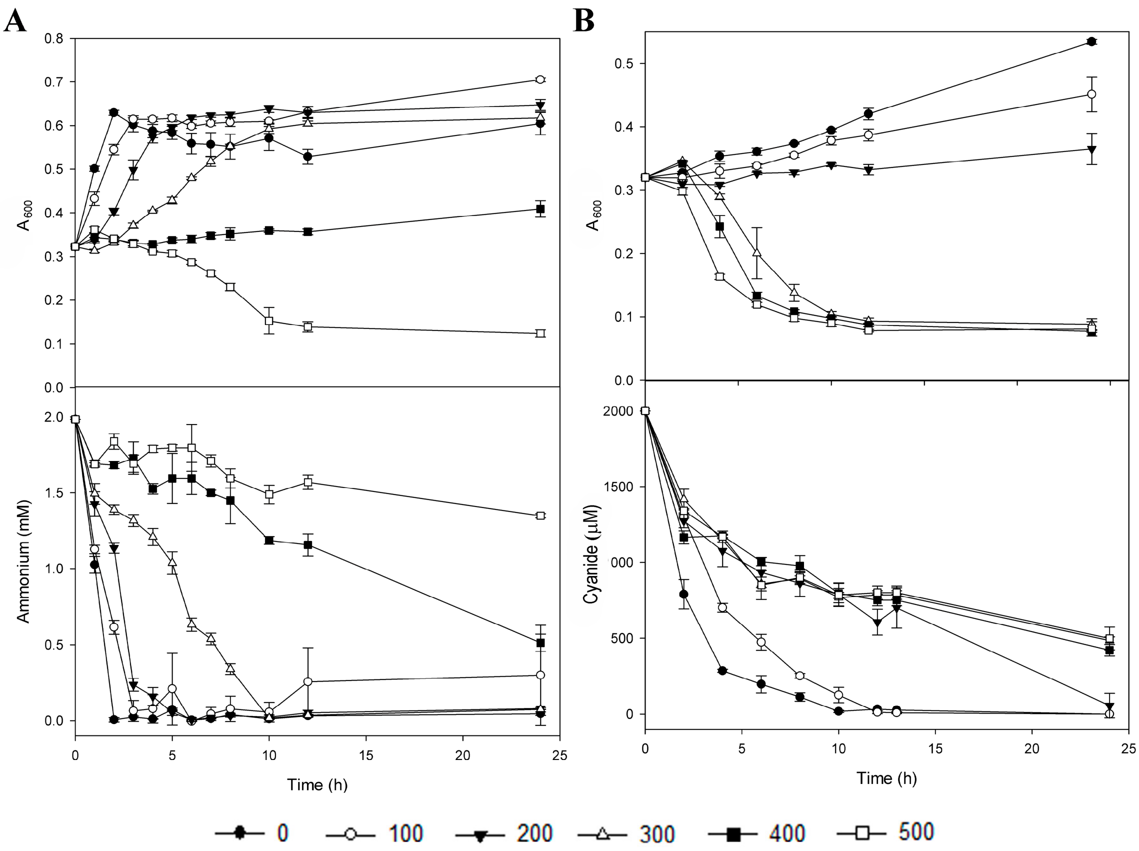

Figure S1. Arsenate tolerance of *P. pseudoalcaligenes* CECT 5344. Cells were grown in M9 minimal medium with 50 mM sodium acetate as carbon source, and 2 mM ammonium chloride (panels A, left) or 2 mM sodium cyanide (panels B, right) as the sole nitrogen source, with different concentrations of arsenate, from 0 to 500 mM. Bacterial growth, upper panels; N-source consumption, lower panels.

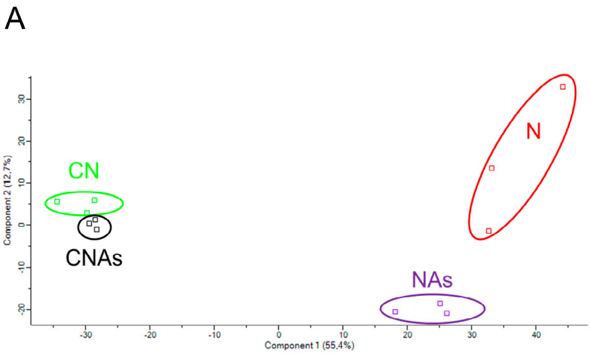

B

Sample	Replicate	# Proteins	# Unique proteins
N	1	924	1177
	2	1105	
	3	1031	
NAs	1	1145	1312
	2	1205	
	3	1204	
CN	1	1446	1591
	2	1480	
	3	1525	
CNAs	1	1506	1581
	2	1511	
	3	1484	

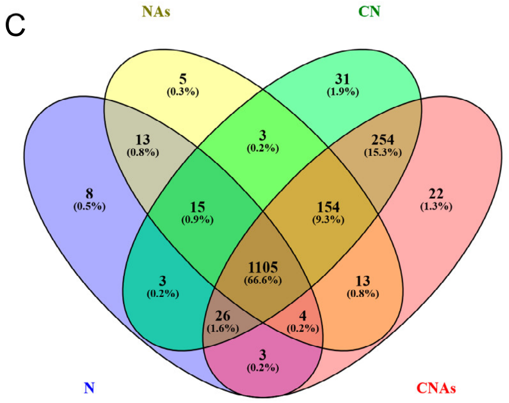

Figure S2. Qualitative proteomic analysis of *P. pseudoalcaligenes* CECT 5344 cells grown with 2 mM ammonium chloride or 2 mM sodium cyanide as nitrogen source, without (N or CN, respectively) or with 0.25 mM arsenite (NAs or CNAs). A) principal component analysis; B) summary of the number of proteins identified per biological replicate and condition; C) Venn diagram of identified proteins.

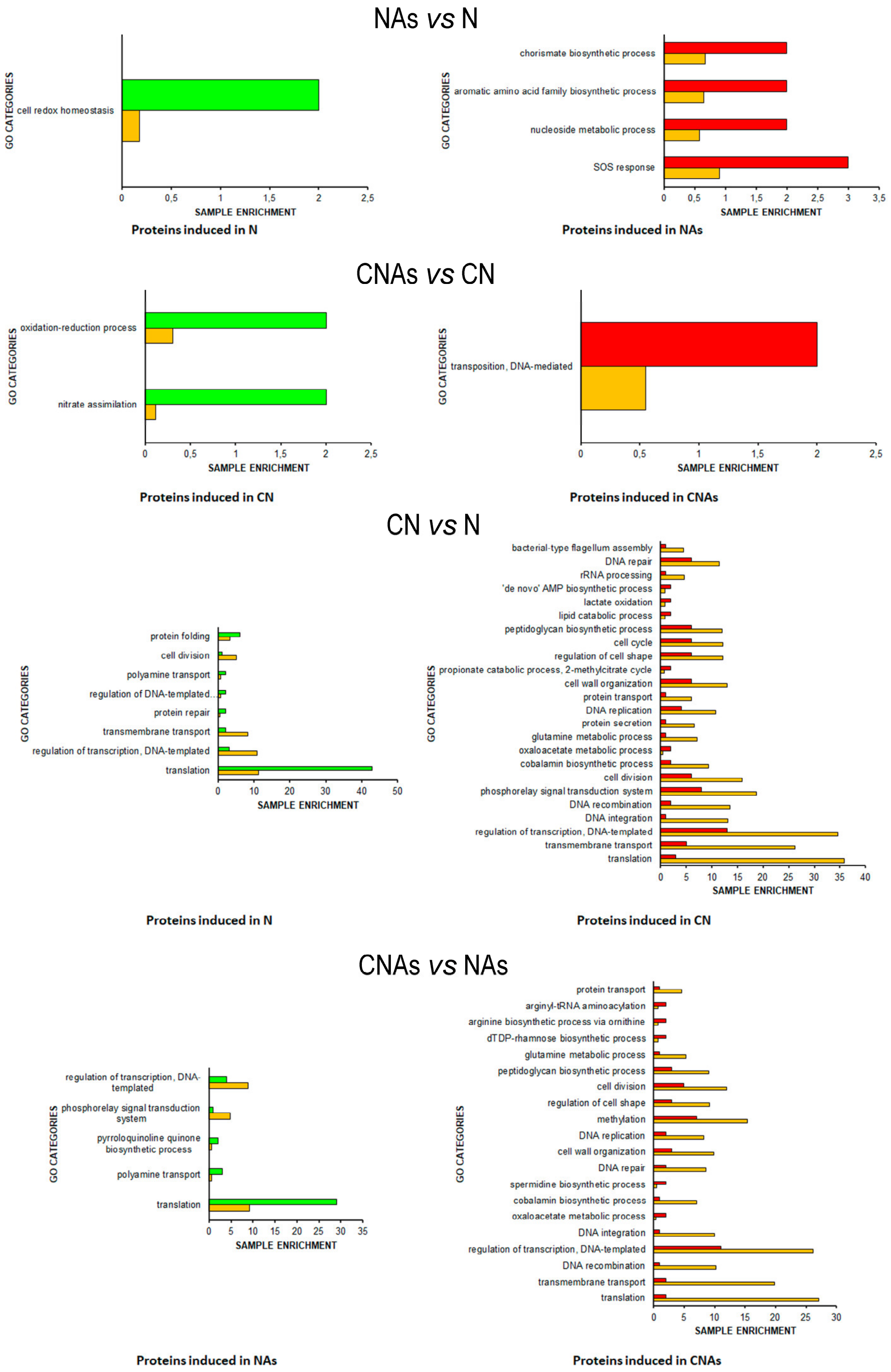

Figure S3. GO enrichment analysis of the different proteomic comparisons. Significative changes in GO groups among proteins over-represented in each condition (green or red) for each comparison are shown. The genome of the wild-type strain was considered as reference (yellow).

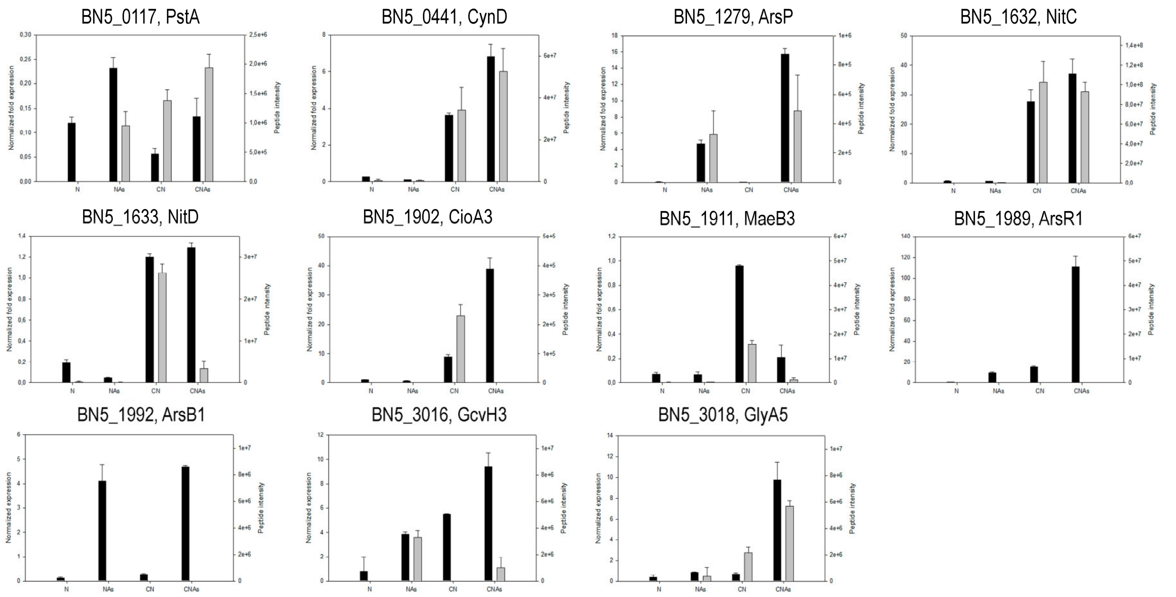

Figure S4. Comparison of the transcriptional and proteomic analyses for some genes of *P. pseudoalcaligenes* CECT 5344 and their products. The relative gene expression determined by qRT-PCR (black bars), and the normalized peptide intensities of its encoded proteins (grey bars), are shown for the different culture conditions: N, ammonium chloride as nitrogen source, without arsenite; NAs, ammonium chloride as nitrogen source, plus 0.25 mM As(III); CN, sodium cyanide as nitrogen source, without arsenite; and CNAs, sodium cyanide as nitrogen source, plus 0.25 mM As(III).
