# Supplementary material for: Proteomic Analysis of Arsenic Resistance during Cyanide Assimilation by Pseudomonas pseudoalcaligenes CECT 5344
Source: Int J Mol Sci. 2023 Apr 13;24(8):7232. doi: 10.3390/ijms24087232 (PMC10138600; doi:10.3390/ijms24087232)
Supplement: Supplementary file 1 [file ijms-24-07232-s001.zip › TableS1_ProteomicSearchParameters_20220120.pdf]

Table S1. Proteomic search and quantification parameters.

Parameter			Data
Specie		*Pseudomonas pseudoalcaligenes* (strain CECT 5344)	
Group specific parameters	Type		**Standard**
	Modifications	Variable modifications	**Oxidation (M), Acetyl (Protein N-term)**
		Fixed modifications	**Carbamidomethyl ©**
	UHPLC system	Model	**Dionex Ultimate 3000 nano UHPLC system**
		Separation column	**Acclaim pepmap C18, 500 nm x 0.075 mm, 2 µm pore size**
		Trapping	**0.2 µg/µL at 10 µL/min flow rate, 5 min**
		Mobile phase	**2% acetonitrile / 0.05% trifluoroacetic acid**
		Gradient time	**60 min**
		Gradient	**5-40 % acetonitrile / 0.1 % formic acid**
		Flow rate	**300 nL/min**
	Instrument	Max. number of modifications per peptide	**3**
		Instrument type	**Orbitrap Fusion**
		First search peptide tolerance	**20**
		Main search peptide tolerance	**4.5**
		Peptide tolerance unit	**ppm**
		Individual peptide tolerance	**yes**
		Isotope match tolerance	**2**
		Isotope match tolerance unit	**ppm**
		Centroid match tolerance	**8**
		Centroid match tolerance unit	**ppm**
		Centroid half width	**35**
		Centroid half width unit	**ppm**
		Time villey factor	**1.4**
		Isotope valley factor	**1.2**
		Isotope time correlation	**0.6**
		Theoretical isotope corretaliton	**0.6**
		Recalibration unit	**ppm**
		Min. peak lenght	**2**

		Min. DIA peak lenght	**1**
		Max. change	**7**
		Min score for recalibration	**70**
		Cut peaks	**yes**
		Gap scans	**1**
		Intensity threshold MS1	**no**
		Intensity threshold MS2	**no**
		Check mass deficit	**yes**
		Intensity determination	**Value at maximum**
		Centroid position	**Gaussian**
		DIA initial precursor mass tolerance (ppm)	**20**
		DIA initial fragment mass tolerance (ppm)	**20**
		DIA corr. threshold for feature clustering	**0.85**
		DIA prec. mass. toll.for feat. clustering (ppm)	**2**
		DIA frag. mass. toll.for feat. clustering (ppm)	**2**
		DIA score N	**7**
		DIA min. score	**1.99**
		DIA quant method	**Mixed LFQ split**
		DIA feature quant method	**Sum**
		DIA top N fragments for quant	**10**
		DIA top msms intensity quantile for quant	**0.85**
		DIA min. Msms intensity for quant	**0**
		DIA precursor filter type	**None**
		DIA min. fragment overlap score	**1**
		DIA min. precursor score	**0.5**
		DIA min. profile correlation	**0**
		DIA global ML	**yes**
		DIA adaptative mass accuracy	**no**
		DIA mass window factor	**3.3**
		DIA background subtraction	**no**
		DIA background subtraction quantile	**0.5**
		DIA background subtraction factor	**4**

		DIA transfer q-value	**0.3**
		DIA LFQ weighted median	**no**
		DIA XGBoost Base Score	**0.4**
		DIA XGBoost Sub Sample	**0.9**
		DIA XGBoost learning objective	**Binary logistic raw**
		DIA XGBoost Min child weight	**9**
		DIA XGBoost Maximum Tree Depth	**12**
		DIA XGBoost Estimators	**580**
		DIA XGBoost Gamma	**0.9**
		DIA XGBoost Max Delta Step	**3**
		DIA no ML	**no**
		DIA only isos for recalibration	**yes**
		DIA min. peaks for recalibration	**5**
	Digestion	Digestion mode	**Specific**
		Enzyme	**Trypsin**
		Max. missed	**2**
		Incubation time	**Overnight**
	Label-free quantification	LFQ	
Global parameters	Sequences	FASTA file	***Pseudomonas pseudoalcaligenes* CECT 5344**
		Uniprot	**UP000032841**
		Include contaminants	**yes**
		Min. peptide lenght	**7**
		Max. peptide mass (Da)	**4600**
		Min. Peptide lenght for unspecific search	**8**
		Max. Peptide lenght for unspecific search	**25**
	Protein quantification	Label min. ratio count	**2**
		Peptides for quantification	**Unique + razor**
		Modifications used in protein quantification	**Oxidation (M), Acetyl (Protein N-term)**
		Discard unmodified counterpart peptides	**yes**
		Advanced ratio estimation	**yes**
	Identification	PSM FDR	**0.01**

		Protein FDR	**0.01**
		Site decoy fraction	**0.01**
		Min. peptides	**1**
		Min. razor + unique peptides	**1**
		Min. unique peptides	**0**
		Min. Score for unmodified peptides	**0**
		Min. score for modificed peptides	**40**
		Min. delta score for unmodified peptides	**0**
		Min. delta score for modified peptides	**6**
		Main search max. combinations	**200**
		Razor protein FDR	**yes**
		Second peptides	**yes**
		Match between runs	**yes**
	Label free quantification	Stabilize large LFQ ratios	**yes**
		Require MS/MS for LFQ comparisons	**yes**
		Advanced site intensities	**yes**
		Top3	**yes**
General parameters		Survey scans of peptide precursors	**From 400 to 1500 m/z**
		Resolution	**120 K (at 200 m/z)**
		Ion count target	**5×10^5**
		Isolation	**1.6 Da with the quadrupole**
		Fragmentation	**CID**
		Normalized collision energy	**35**
		Rapid scan MS	**yes**
		Samples for MS2	**Precursors with 2-5 charge state**
		Run mode	**Top speed, with 3 s cycles**
		MS2 spectra search	**MaxQuant software v1.5.7.4**
		Differential expression analysis	**Perseus software v1.6.12.1**
		Criteria for protein identification	**At least 2 peptides**
		Criteria for protein quantification	**Present in 3 replicates**
		Criteria for exclusively expressed proteins	**Present in at least 2 replicates of a condition exclusively**

	Replicates	**3**
	Conditions	**4 (N, NAs, CN, CNAs)**
	Differential expression if	**Fold change ≥ 2 and *p* value < 0.05**
