# Supplementary material for: Proteomic Analysis of Arsenic Resistance during Cyanide Assimilation by Pseudomonas pseudoalcaligenes CECT 5344
Source: Int J Mol Sci. 2023 Apr 13;24(8):7232. doi: 10.3390/ijms24087232 (PMC10138600; doi:10.3390/ijms24087232)
Supplement: Supplementary file 1 [file ijms-24-07232-s001.zip › TableS2.PrimerUsed.pdf]

Table S2. Primers used in this study.

Locus	Protein name	Forward (5'-3')	Reverse (5'-3')
**BN5_0117**	Phosphate transport system permease protein	CCGAACGCCATGAGCACCGTCGACA	AAGATCGCCGGGAAGATACCGCCTT
**BN5_0252**	ArsR family transcriptional regulator	ACGCCGCCGGGCAACTGCTCAAG	CTCGCTGACGTTGCGCTCGCCTT
**BN5_0255**	Hypothetical protein	CGCCGCAGGTCTGATTCTTATCGC	GGCACCACACCGATCCAACCCCA
**BN5_0258**	C-N hydrolase, nitrilase/cyanide hydratase (AguB)	GACGGCCCCGGCTACCACGAGAA	ATCCCAGCAGATGCCGACACCGAT
**BN5_0441**	Nitrate/nitrite transport system ATP-binding protein	TACGTATCGACCTGCCACGGCCAC	AGCGAGCGGTTCACCAGAAAGTCCA C
**BN5_1354**	Sulfite reductase (NADPH) hemoprotein β-component	CGGTTACGGGCACTTCACCACACGA C	ATGCATATCGTGCTCGGCCAACCAG
**BN5_1632**	Nitrilase NitC	TGAGCGTATGGTCTGGGGGCAGGG	CGCCGCATGAATCTGCTCGCCATC
**BN5_1633**	Radical SAM domain-containing protein NitD	CCGTGGTGCTGCCATCCTTTGCGAAT	CAAGCGGCGAAACCAGGCGTCCTC
**BN5_1902**	Cytochrome *bd* ubiquinol oxidase subunit I	GGCCTTCCTGCTGGCGATCA	GCGGCGCACCGAAGATGTCA
**BN5_1911**	MeaB3, malate dehydrogenase	CCCGCCAACGTCTTCCGCTTCACC	AAGCGCACCGATGTTTCCCAGTCCA
**BN5_1989**	ArsR family transcriptional regulator (ArsR1)	ACGTGGCCCGCAACACCTTGTCCT	CGGCCCTGCTGCTCAATCGAGACCA
**BN5_1991**	Arsenate reductase (thioredoxin), ArsC1	AGCCATCCGAAGGGCGCAGTGCAT	GGCGCACCGGGCACCACGAACTC
**BN5_1992**	Arsenite transporter, ArsB1	CGTGTTCCAGGCCATCGGTCGCAT	TTGGCCCAGCGCACCGAAGTCCAC
**BN5_1993**	Arsenate reductase (glutaredoxin), ArsC2	AAGACGCCACCCGACCGCGAGAC	CGCATCGCTCCACTTCGGGTCGTCA
**BN5_2541**	23S rRNA (cytidine2498-2'-O)-methyltransferase	CATCGCCTGGTTGTCCGCCTC	CGGCAAACTGGTGGACGACGC
**BN5_2707**	ArsR family transcriptional regulator (ArsR2)	ATGCCATGTCGCCAACCGAAGTCT	CGCCTCTTCCAGTGCCGAGGTCA
**BN5_2708**	Arsenate reductase (thioredoxin), ArsC3	TTTTCTTTGGCCCGGCGACTCGTT	CGCTCACTCGCTTCTGGATCTGCT
**BN5_2786**	Arsenate reductase (glutaredoxin), ArsC5	TGCGCTATCTGGAAACGCCACCC	CTCGCCGCTACGCAGCAGGT
**BN5_2838**	Arsenate reductase (glutaredoxin), ArsC4	ACGAGCATGGCTGGCAGACCAT	ATCGGCTTTCTGCGCGTCGTC
**BN5_3016**	Glycine cleavage system H protein	CTGGTTACCGTCGGCATCACCCA	ACTTGGCTTCCGGCAGTTGCAC
**BN5_3018**	Glycine hydroxymethyltransferase	ACTGCTCAATGCGGGCGACACCA	AGCTTGCCCGAGGACGACACCTT
**BN5_3703**	RpoB, DNA-directed RNA polymerase subunit beta	AGCTGCTGCGTGCGATCTTCGGTGAG A	CCAATTGCTCGTTCAGGGCGTCGTCA G
